# Neuromodulation of prefrontal cortex promotes deep processing during language comprehension: a tDCS/EEG study

**DOI:** 10.3758/s13415-025-01337-6

**Published:** 2025-08-13

**Authors:** Megan A. Boudewyn, Cameron S. Carter

**Affiliations:** 1 Department of Psychology, University of California, 1156 High Street, Santa Cruz, CA 95064 USA; 2 University of California, Irvine, CA USA

**Keywords:** Language comprehension, EEG, N400, P600, TDCS, Prefrontal cortex

## Abstract

In this study, we used transcranial direct current stimulation (tDCS), a noninvasive neuromodulation technique, to test a set of hypotheses about the extent to which the prefrontal cortex (PFC) contributes to revision and updating processes during language comprehension. Following 20 min of PFC-targeted, Active Control, or Sham tDCS, EEG was recorded while participants performed a widely used paradigm in which they read sentences containing plausible and implausible thematic roles (e.g. The actress/film-maker was directed by the film-maker/actress on set). This linguistic manipulation allowed us to examine comprehension under conditions when shallow processing and deeper processing yield conflicting meaning representations, which previous work has shown often engages revision and updating processes upon detection of the conflict. A different pattern of event-related potential responses was elicited when the same participants encountered implausible thematic roles during reading after receiving Sham compared with PFC-targeted tDCS. Specifically, N400 effects were found after Sham tDCS, whereas robust P600 effects were found after PFC-targeted tDCS (and to a significantly lesser extent, after Active Control tDCS). This suggests that while readers tended to treat implausible thematic roles as semantic anomalies after Sham tDCS, those same readers were more likely to detect conflict and engage in revision and updating in response to implausible thematic roles when in a state of heightened PFC stimulation. These results provide a novel demonstration of within-individual variability in language processing depending on current neurocognitive state and have implications for psycholinguistic theory about PFC contributions to revision and updating processes during language comprehension.

## Introduction

Language comprehension requires navigating complex input very quickly. To explain this, many modern accounts of language processing propose a “multiple stream” approach in which incoming words in context are both shallowly processed (using heuristics and local word-level information) and also processed more deeply (using syntactic rules and the larger context) (Brouwer et al., 2017; Eddine et al., 2024; Kuperberg, 2007; Kuperberg et al., 2020; Li & Futrell, 2024). Event-related potential (ERP) research has been instrumental in motivating these accounts, informed largely by two well-studied ERP components: the N400 and the P600.

The N400 is considered to be a reliable marker of semantic processing during language comprehension (Kutas & Federmeier, 2011; Swaab et al., 2012) It is a negative-going deflection that peaks around 400 ms post word onset, and its amplitude increases in response to a variety of factors that lead to greater demands on semantic processing (e.g., word frequency, word predictability). In contrast, the P600 (a positive-going deflection that peaks after the N400 time window) is elicited by challenging syntactic structures or syntactic errors and is thought to belong to a family of late positivity ERP effects associated with the need to revise or update a developing mental representation (Kaan & Swaab, 2003; Osterhout et al., 1997; Swaab et al., 2012). The distinction between the N400 and the P600 as markers of relatively early semantic processing versus later revision processes has proven useful in detangling the underlying mental operations involved in processing complex language input.

For example, ERP studies that have examined how readers process sentences containing unexpected words have found qualitative differences in the response to fully unexpected words (e.g., for breakfast the boys would plant, in which “plant” has no support from context) compared with words that have some level of support from context (e.g., for breakfast the eggs would eat, in which “eat” is strongly related to the context and revision might render the sentence plausible, such as if the eggs were being eaten). Namely, N400 effects are consistently found in response to the former, suggesting that fully implausible input is typically processed as a semantic anomaly (Hagoort et al., 2004; Kuperberg et al., 2003; Kutas & Federmeier, 2011; Kutas & Hillyard, 1980). In contrast, a large number of studies have found P600 effects in response to implausible words, such as “eat” in “for breakfast the eggs would eat,” which suggests that this type of sentence triggers “deeper” processing, and specifically revision and updating processes (Chow et al., 2018; Hoeks et al., 2004; Kim & Osterhout, 2005; Kolk et al., 2003; Kuperberg et al., 2003; Van Herten et al., 2005, 2006). The fact that the second type of implausible input seems to engage deeper processing than the first implies that readers/listeners may routinely compute mental representations of sentence meaning based on loose semantic relations as well as based on the syntactic structure of the sentence (Kuperberg, 2007). If this was not the case, readers/listeners would process sentences, such as “for breakfast the boys would plant” and “for breakfast the eggs would eat” in the same way, as unexpected input, which should be accompanied by increased N400 amplitude.

Much of the work in this literature has sought to characterize the conditions under which implausible words trigger revision and updating processes, reflected by the presence of P600 effects. This has provided detailed information about the types of mental representations that readers/listeners likely compute during comprehension. For example, initial reports of P600 effects elicited by implausible-but-somewhat-contextually-supported words involved sentences in which reversal of the roles assigned each word (thematic roles) would render the sentence plausible, such as in “At breakfast, the eggs would eat…” (rendered plausible if eggs are assigned the role of the object of the action instead of the agent of the action) (Kolk et al., 2003; Kuperberg et al., 2003). Later work tested nonreversible implausible sentences, such as “The meals were devouring…” to show that attempts at revision to implausible-but-somewhat-contextually-supported words seem to be made even if the sentence cannot be “fixed” by simply reversing thematic role assignment (Kim & Osterhout, 2005). Interestingly, N400 effects in response to implausible thematic role assignment have sometimes been observed, particularly under conditions that promote relatively shallow processing, such as tasks that require minimal attention, very challenging syntactic structures in which syntactic parsing may fail, and speeded stimulus presentation rates (Chow et al., 2018; Kolk et al., 2003; Liao et al., 2022; Van Herten et al., 2006).

Overall, the consistent reports of P600s in response to sentences containing implausible thematic role assignment have contributed significantly to influential cognitive neuroscience models of language processing by demonstrating that these types of sentences often trigger revision and updating processes. However, the studies that have found N400s in response to these sentences suggest that this is not always the case. In addition, some studies of individual differences in language processing have found that some individuals tend to adopt a “revision-forward” strategy (also called “P600 dominant”), whereas other individuals tend to process sentences, such as those used in the current study, as semantic anomalies (also called “N400-dominant”) (Coderre & Cohn, 2024; Kim et al., 2018; Kos et al., 2012; Nakano et al., 2010; Tanner, 2019; Tanner & Van Hell, 2014). In the current study, we were interested in examining differences in processing strategy that might occur *within* an individual. Thus, we examined ERP responses in the same participants, keeping the experimental manipulation and task constant, and manipulating brain state within participant using transcranial direct current stimulation (tDCS), a mild, noninvasive method of neural stimulation. In short, the goal of the current study was to use ERPs to test the extent to which a reader engages in deeper processing is determined by current neurocognitive state, and specifically on the current state of the prefrontal cortex (PFC).

We focused on the PFC in particular for two reasons. First, as noted above, the greatest disparities in the pattern of results that have been obtained in studies of this sentence type (i.e., obtaining N400 instead of P600 effects) seem to be related to differences across studies that may have changed attention and cognitive control demands, which are both strongly linked to PFC circuits. Second, while the neural generators of the P600 are not known, some work has suggested that a set of neural generators in the PFC may contribute to the P600 and related late positivity effects (Brouwer & Hoeks, 2013; Friederici et al., 2003; Van de Meerendonk et al., 2011). Source localization work has also suggested that the anterior cingulate (medial PFC) may contribute to the P600 (Shen et al., 2016), which would fit with recent work that has correlated the P600 with individual differences in performance on general cognitive control tasks (Brothers et al., 2022), as medial PFC is thought to support domain general cognitive control and conflict detection processes (Botvinick et al., 2004; Carter & Van Veen, 2007; Carter et al., 1998; Van Veen & Carter, 2006). P600 effects elicited by implausible thematic role assignment have also been shown to be correlated with individual differences in performance on cognitive control and working memory tasks (Li et al., 2023; Ye & Zhou, 2008).

In the current study, we sought to test a neurocognitive account of language processing in which current PFC state can influence the extent to which individuals engage in revision and updating processes during language comprehension. We used transcranial direct current stimulation (tDCS) to administer mild, noninvasive neural stimulation to the PFC before participants completed a language comprehension task in which they read sentences featuring plausible or implausible thematic role assignment (e.g., *The actress/film-maker was directed by the film-maker/actress on set*.). Event-related potential responses after PFC-targeted tDCS were compared with those recorded after Active Control tDCS and Sham (placebo) tDCS in a within-participants design. The use of an active control condition allowed us to test whether any observed tDCS effects could be attributed to stimulation of the PFC specifically or to broad effects of neural stimulation.

To the extent that the PFC promotes relatively deep processing, and specifically revision and updating processes, during language processing, we expected to see evidence for relatively deeper processing in the PFC-targeted tDCS condition compared with either Active Control or Sham tDCS. Relatively deep processing would be indicated by the presence of a P600 in response to implausible thematic role assignment, whereas relatively shallow processing would be indicated by a reduced P600 effect or by N400 effects. This pattern would demonstrate variability *within* an individual in how language is processed as a function of current neurocognitive state; in other words, it would show that readers do not always process language the same way, which is not accounted for by most language processing theories. It would also specifically implicate PFC circuits in promoting revision and updating processes during language comprehension. Instead, if equivalent ERP effects were obtained, this would suggest that readers obligatorily generate mental representations of sentence meaning based on the syntactic structure of the sentence and also based on loose semantic relations.

## Methods

### Participants

This experiment received research ethics committee approval from the UC Davis and UC Santa Cruz institutional review boards. Seventy participants were recruited and enrolled at the University of California, Davis during an initial informed consent appointment that was conducted remotely. Of those, 59 came into the lab and completed at least one experimental session (tDCS + EEG) (40 females; 19 males; 0 nonbinary; average age: 25, range 18–49 years). Data from all participants who completed at least one in-person experimental session was included in the final analyses. As testing order was counterbalanced with respect to tDCS protocol condition, participant drop-out in testing sessions 2 and 3 was randomly distributed across the three tDCS protocol conditions. This yielded a dataset of 56 active PFC stimulation sessions, 56 sham stimulation sessions, and 54 active control stimulation sessions. Following EEG processing (see below for details), the final sample consisted of 50 sessions in each tDCS protocol condition with usable EEG data. All participants were right-handed native speakers of English. Participants were also screened for contraindications to tDCS, such as a history of epilepsy or metal in the skull. Participants were compensated at a rate of $20 per hour and also received a $50 bonus if all sessions were completed.

### Protocol overview

**Fig. 1:**
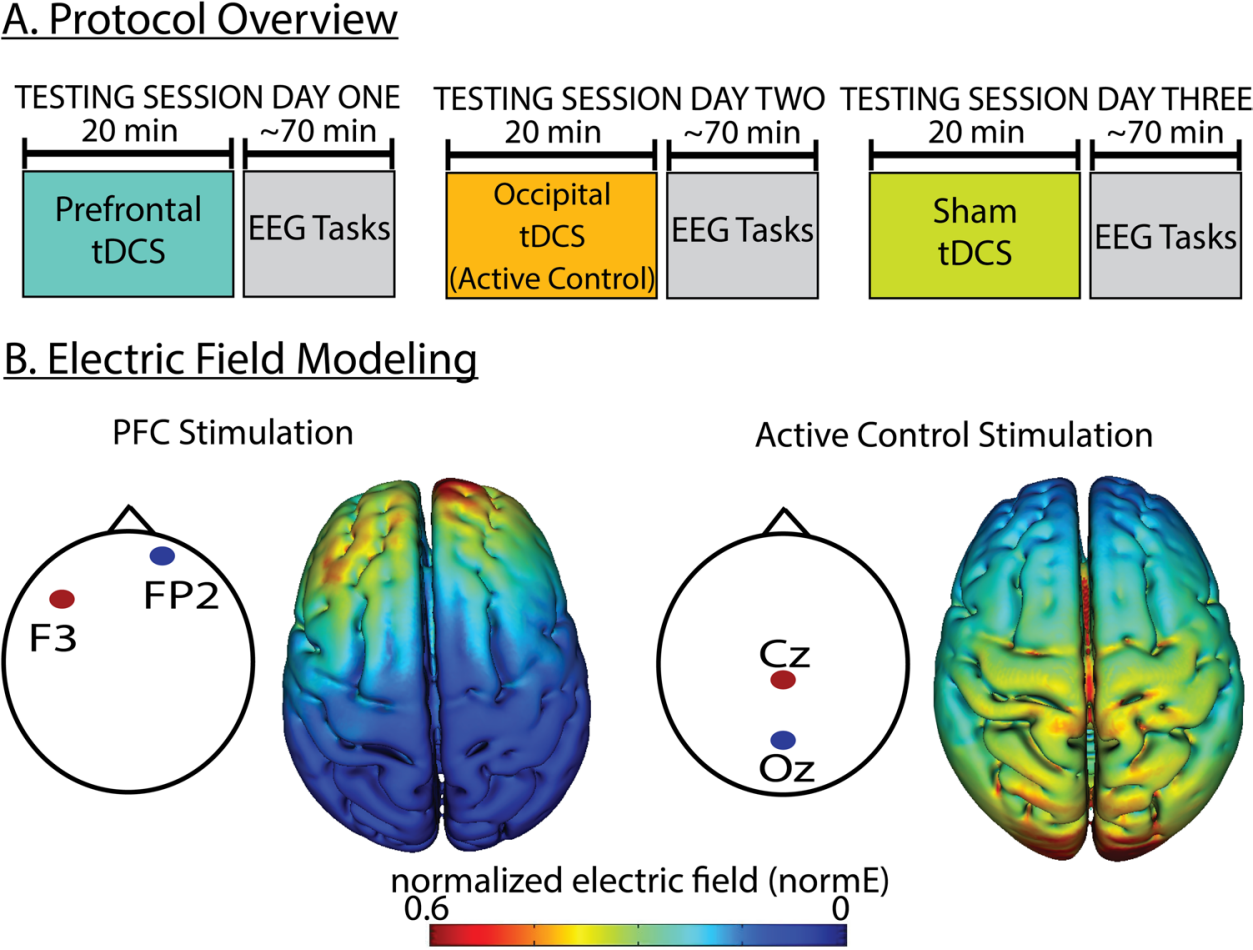
Protocol overview and electric field modeling of PFC-targeted and Active Control tDCS. **A** This study used a double-blind crossover within-participants study design. Participants were randomly allocated to testing order. **B** Anode (red) and cathode (blue) locations for PFC-targeted tDCS and Active Control tDCS montages. Normalized electric field produced by montage, projected onto 3D rendering of brain in MNI space

This study used a double-blind, crossover, within-participants design. Participants completed an initial consent and task practice session and three total experimental sessions, each consisting of 20 min of tDCS following by about 1 h and 10 min of cognitive tasks while EEG was recorded (see Fig. 1 for an overview of the study design). Participants completed two ~ 20-min cognitive control tasks prior to completing the ~ 30 min language task that was the focus of the current study; data from these other tasks was not analyzed here. Participants completed active PFC stimulation, active control stimulation and sham stimulation sessions on different days; session order was randomly assigned. The average number of days between sessions was 18 (range 2–147 days). As a note, data collection started in the context of the ongoing Covid-19 pandemic, which contributed to the variability in session timing.

All electrodes (EEG and tDCS) were prepared prior to the administration of tDCS, to reduce delay between completion of tDCS and EEG task onset. In addition, all possible tDCS stimulation electrodes were prepared during each session in order to maintain experimenter blinding. During tDCS administration, participants completed the Dot-Pattern Expectancy (DPX) task, which is a variant of the AX-Continuous Performance task (AX-CPT), both of which have been shown in a number of fMRI studies to engage prefrontal cognitive control circuits (Henderson et al., 2012; Jones et al., 2010; Lopez-Garcia et al., 2016; MacDonald III et al., 2007). Participants completed the DPX task during stimulation in order to promote cognitive control engagement, as there is some work suggesting that combining tDCS with a task that engages the targeted neural circuits enhances the effects of tDCS as compared with stimulation while at rest (Andrews et al., 2011). Participants then completed the language comprehension task. Both tasks were presented using Neurobehavioral Systems Presentation (www.neurobs.com).

At the end of each session, participants also made a guess as to whether they thought that the day’s session had been active or sham stimulation. This was done to assess the effectiveness of the blinding procedure. Participants were close to chance at correctly guessing the stimulation condition, correctly guessing 46.6% of the time.

### DPX task (During Stimulation)

During stimulation, participants completed the DPX task, which, as noted above, is a variant of the AX expectancy task in which dot-patterns are used as cue-probe pairs rather than letters. The version we used is freely available online (http://cntracs.ucdavis.edu/dpx). Participants were presented with 144 trials across 4 blocks of 36 trials each, in four conditions: AX (72%), AY (11%), BX (11%), and BY (6%). AX trials (dot-pattern “X” when preceded by dot pattern “A”) represent targets; all cues and other cue-probe combinations represent nontargets. Following from previous work with both the AX-CPT and DPX versions of this task, we used d prime context as a behavioral measure of cognitive control (Lesh et al., 2013; Lopez-Garcia et al., 2016; Smucny et al., 2018). D prime context was calculated by subtracting BX false alarms from AX hits (Cohen et al., 1999).

### Language task (Poststimulation)

Stimuli consisted of sentences presented visually, one word at a time in rapid serial visual presentation (RSVP) format (300 ms duration per word, 200 ms interstimulus interval).

Experimental sentences featured either plausible (e.g., The actress was directed by the film-maker on set) or implausible (e.g., The film-maker was directed by the actress on set) thematic role assignment. Critical words were always animate; no sentences featured semantic violations (i.e., all sentences described possible scenarios, as in the example above). Additional example stimuli are included in the supplemental materials.

A total of 216 experimental sentence sets were created. Items were then distributed across three lists such that within a list, each item only appeared once and in one of the two experimental conditions. Each participant completed one list at each experimental session, with list order assigned randomly. Therefore, each participant read 36 unique items per condition per session without repetition. Ninety sentences with an unrelated experimental manipulation (semantic predictability) were included and serve as fillers for the current study. Comprehension questions that directly asked about thematic role assignment (e.g., Who was directed? A) the actress or B) the film-maker) were included to encourage attentive reading.

### tDCS administration and EEG recording

tDCS was administered and EEG was recorded by using a StarStim32 neurostimulator (neuroelectrics.com). The StarStim32 system includes both recording and stimulation electrodes. Recording electrodes had a π cm^2^ circular contact area based on an Ag/AgCI pellet with a 12-mm diameter. For PFC-targeted tDCS, anode placement was over left dorsolateral PFC (F3) and cathode placement was at a right supraorbital site (FP2). This montage is commonly used to target the dorsolateral PFC (Laakso et al., 2016). Anode placement in our Active Control montage was at a central electrode site (Cz) and cathode placement was over occipital cortex (Oz). The Active Control montage was selected in order to provide maximal contrast to the PFC-targeted montage, as electric field modeling indicates that the two protocols produce distinct patterns of electric modulation in cortex, as can be seen in Fig. 1. HD-Explore (soterixmedical.com) was used to generate the electric field models, using an MNI standard brain template. All four stimulation sites were prepared at each session so that experimenters remained blind as to which tDCS protocol was administered during each session. During PFC-targeted and Active Control stimulation, current was administered for 20 min at 2 mA, with a 30-s ramp-up and ramp-down. Sham stimulation followed the same procedure, except that current ramped down and remained off after the 30-s ramp-up at the beginning of the 20-min period. These parameters were selected based on our previous tDCS work (Boudewyn et al., 2019, 2020).

The EEG signal from the 32-channel StarStim system was recorded with a 1,000 Hz sampling rate and a 0–200 Hz bandpass filter. Impedances were kept below 5 kΩ. The right mastoid served as the recording reference. One scalp electrode (T7) was used to record vertical EOG rather than as a scalp EEG channel to enable the recording of eye movements. A bipolar vertical EOG channel was calculated offline using this below-eye channel and FP1 (above-eye).

### EEG preprocessing and preparation for analysis

The EEG data were preprocessed using the EEGlab Toolbox for MATLAB (Delorme & Makeig, 2004) and the ERPlab plugin (Lopez-Calderon & Luck, 2014). The EEG data were high-pass filtered using a noncausal Butterworth filter (half-amplitude cutoff of 0.05 Hz, 12 dB/octave slope). The data were screened for nonfunctioning channels, which were defined as channels with unusable data during one-third or more of the total recording time. Independent component analysis (ICA) was used to correct for eye blinks and horizontal eye movements using the approach described in (Boudewyn et al., 2023). Nonfunctioning channels were excluded from the ICA and then interpolated using a spherical spline algorithm. Then, the preprocessed continuous EEG data were segmented into epochs of − 800 to 3,500, time-locked to the auxiliary verb onset of trials on which participants responded correctly to the comprehension question. Following segmentation, epochs were screened for any remaining artifacts, using ERPlab functions for detecting extreme values (default set to − 150 to 150 uV). On average, 19.44% of trials were rejected due to the presence of artifacts, with an average of 29 trials per condition remaining in each dataset (range 19–36).

### Data analysis

To examine thematic role plausibility effects, we examined single-trial mean amplitudes in the N400 and P600 time windows corresponding to each word in the critical region, which included the auxiliary verb, verb and by-phrase (by + determiner + NP2). Relatively long-epochs time-locked to the onset of the auxiliary verb were used to calculate the mean amplitude in the N400 (300–500 ms post word onset) and P600 (500–800 ms post word onset) time windows corresponding to each word in this region. This allowed us to use a preauxiliary verb baseline period for the entire sequence (− 200 to 0 ms). This was done to avoid the possibility of introducing a false effect for words appearing later in the region if the − 200 to 0 ms period before those words contained an effect from the previous word. For example, an N400 or P600 effect in response to the verb would overlap in time with the − 200 to 0 window time-locked to the next word (the start of the by-phrase), which therefore not serve as a suitable baseline window for the verb; the use of a preauxiliary verb baseline for all analyses prevented this problem.

## Results

All statistical analyses were performed in R (R Core Team, 2013) on single-trial data using the lme4 package (Bates et al., 2015). Results are summarized below and full model output as well as corresponding R code and details about model selection is provided in the supplemental materials.

### Behavioral data: DPX task

**Fig. 2:**
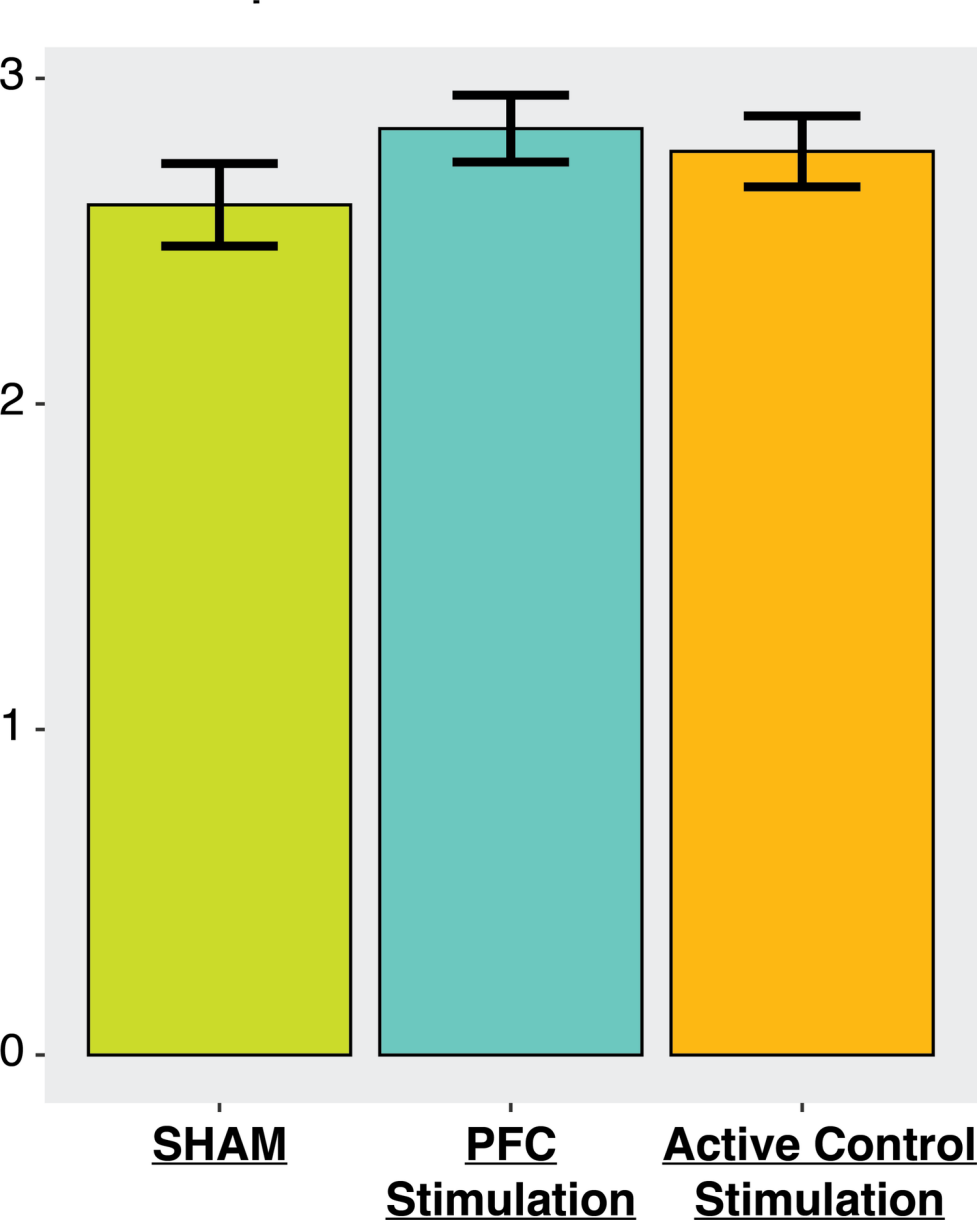
Behavioral results from DPX task

Participants completed the DPX task during tDCS administration in all protocol conditions. The DPX provides a well-validated behavioral measure of cognitive control, d prime context. We examined the influence of tDCS protocol on d prime context using a linear mixed effect model, with Sham stimulation as the reference level and a random intercept for participants. As illustrated in Fig. 2, d prime context was significantly increased following PFC tDCS compared to Sham (β = 0.17773, SE = 0.06923, *p* = 0.0108). In contrast, the difference in d prime context for Active Control tDCS compared with Sham was not statistically significant (β = 0.10357, SE = 0.06931, *p* = 0.1363).

### Behavioral data: Language task

As noted above, comprehension questions were included on the language task in order to encourage attentive reading, and we expected that comprehension question accuracy would be similarly high across plausibility conditions and tDCS protocols. As expected, behavioral performance on the comprehension questions was high in all conditions and protocols, ranging between 93.9% and 94.95% correct. We did not find evidence for statistically significant differences in accuracy as a function of plausibility (β =  − 0.763, SE = 0.8007, *p* = 0.342) or tDCS protocol (PFC tDCS vs. Sham: β =  − 0.394, SE = 0.2896, *p* = 0.625; Active Control tDCS vs. Sham: β = 0.29, SE = 0.8122, *p* = 0.722) when we examined accuracy using a linear mixed-effect model (with Sham stimulation and the plausible condition as the reference levels and a random intercept for participants).

### EEG data step 1: Testing for overall effect of tDCS protocol on ERP plausibility effects

**Fig. 3:**
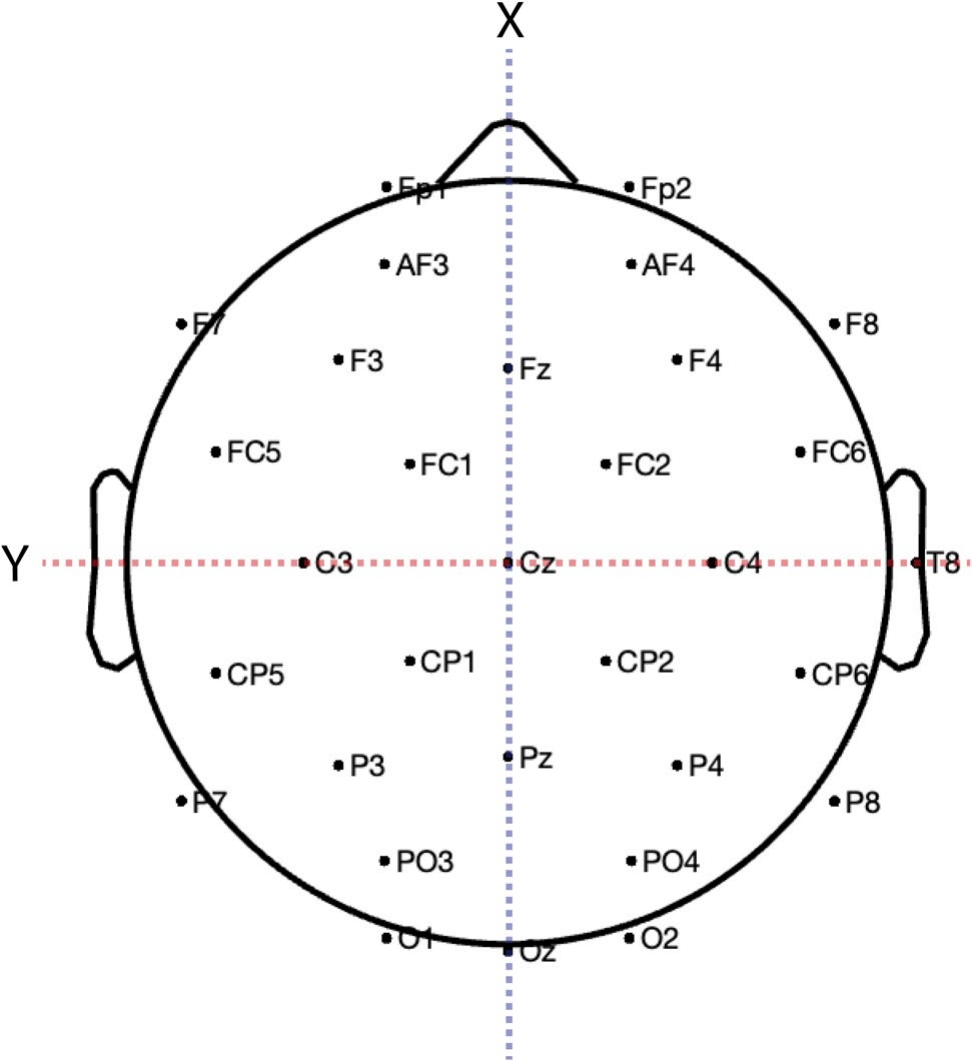
Electrode channel layout. Scalp distribution coordinate system displayed with dashed lines. The X-axis (dashed blue) captures the anterior-to-posterior plane, passing through the nasion. The Y-axis (dashed red) captures the left-to-right plane, passing through the pre-auricular points. The Z-axis (not pictured) captures the superior-to-inferior plane, passing through the vertex (Cz)

We first examined ERP effects of plausibility and whether any potential plausibility effects varied as a function of tDCS protocol by using a set of linear mixed-effects regression models with fixed effects of tDCS protocol (Sham, PFC-Targeted, Active Control) and condition (Plausible, Implausible), and the X, Y, and Z coordinates of each electrode (Boudewyn et al., 2024; Winsler et al., 2018, 2023). Specifically, an electrode’s X-dimension value captured its position along the anterior-to-posterior axis, whereas the Y-dimension captured the left-to-right axis and the Z-dimension captured the superior-to-inferior axis corresponding to electrode location (Fig. 3). Condition was treatment-coded using Plausible as the reference condition. tDCS protocol was also treatment-coded, using Sham as the reference condition. The model intercept therefore reflects the reference levels of tDCS protocol (Sham) and condition (Plausible).

We did not expect that ERP effects would necessarily be present or equivalent at all electrode locations, and so we also included interaction terms between X–Y-Z coordinates and condition to capture the scalp distribution of any observed effects of condition. Significant main effects of scalp distribution are therefore not interpretable other than to indicate that overall voltage values varied across electrodes. However, significant interactions of scalp distribution variables and condition are interpretable. For example, an interaction between condition and the X-distribution predictor would capture a difference between conditions that was maximal at anterior electrode sites. Finally, we included an interaction term between tDCS protocol and condition. The random effects structure for each model included a random intercept for participants and by-participant random slopes for the effect of condition.

Because ERPs in both the N400 and P600 time windows with respective to multiple word positions in the sentence were examined (2 time windows X 5 word positions), it was necessary to account for multiple comparisons when assessing the significance of any observed effects. We therefore set the uncorrected significance level to *p* < 0.005 to achieve a Bonferroni-corrected alpha of 0.05 (0.05/10 = 0.005).

The primary purpose of this initial test was to establish whether tDCS protocol condition significantly influenced ERP effects of condition; in other words, the key question tested by this set of models concerned the plausibility condition by tDCS protocol interaction. The results of this test are summarized in the “Step 1: Testing for Overall Effect of tDCS Protocol on ERP Plausibility Effects” section of Table 1. The results showed significant interactions of tDCS protocol and condition starting in the P600 time window relative to the main verb and extending through all subsequent time windows until the first NP2 time window (verb + by + the + NP2; e.g., *directed by the actress/film-maker*) (Table 1).

**Table 1:** Summary of statistical results involving effects of tDCS protocol and plausibility condition

	Auxiliary verb		Verb		“by”		“the”		“NP2”	
	N400	P600	N400	P600	N400	P600	N400	P600	N400	P600
Step 1: Testing for Overall Effect of tDCS Protocol on ERP Plausibility Effects										
tDCS by plausibility interaction?	ns	^	^	***	***	***	***	***	***	^
Step 2: Testing Pairwise Comparisons of tDCS Protocol on ERP Plausibility Effects										
PFC Stim vs. Sham										
tDCS by plausibility effect?	–	–	–	***	***	***	***	***	***	–
Active Control vs. Sham										
tDCS by plausibility effect?	–	–	–	***	***	***	***	***	***	–
PFC Stim vs. Active Control										
tDCS by plausibility effect?	–	–	–	^	^	***	***	ns	ns	–
Step 3: Testing for ERP Plausibility Effects in each tDCS Protocol Separately										
Sham										
*Plausibility*	–	–	–	ns	ns	ns	ns	ns	ns	–
*Plausibility x X*	–	–	–	***	***	***	***	***	***	–
*Plausibility x Y*	–	–	–	^	ns	ns	ns	ns	^	–
*Plausibility x Z*	–	–	–	ns	ns	^	ns	^	***	–
PFC Stim										
*Plausibility*	–	–	–	ns	ns	ns	ns	ns	ns	–
*Plausibility x X*	–	–	–	***	***	***	***	***	***	–
*Plausibility x Y*	–	–	–	***	***	***	***	***	***	–
*Plausibility x Z*	–	–	–	***	ns	ns	***	***	***	–
Active Control										
*Plausibility*	–	–	–	ns	ns	ns	ns	^	ns	–
*Plausibility x X*	–	–	–	^	ns	^	***	ns	ns	–
*Plausibility x Y*	–	–	–	ns	^	***	***	***	***	–
*Plausibility x Z*	–	–	–	***	***	***	***	***	^	–

See supplemental materials for full model output. ns = not significant; ^*p* < 0.05; ***p < 0.005 (corrected significance level)

### EEG data step 2: Testing pairwise comparisons of tDCS protocol on ERP plausibility effects

Given that the “Step 1” analysis revealed that there were significant interactions between tDCS protocol and plausibility condition (when all three tDCS protocols were included in the model), we next sought to identify which of the three tDCS protocols significantly differed from each other. We used a set of pairwise comparisons examining the extent to which ERP effects of plausibility varied as a function of tDCS protocol, for each unique pair of tDCS protocols. Models were otherwise identical to those described above.

As summarized in Table 1, ERP Plausibility effects following PFC-targeted tDCS significantly differed from those that followed Sham tDCS in the same time windows indicated by the “Step 1” test (verb + by + the + NP2; e.g., *directed by the actress/film-maker*). Event-related potential Plausibility effects following Active Control tDCS also significantly differed from those that followed Sham tDCS in the same time windows. Event-related potential Plausibility effects following PFC-targeted stimulation also significantly differed from those that followed Active Control stimulation, although this difference was limited to a portion of the by-phrase only.

### EEG data step 3: Testing for ERP plausibility effects in each tDCS protocol separately

Finally, based on the results of the “Step 2” analyses, which indicated significant differences in ERP Plausibility effects as a function of tDCS protocol for all protocol pairs, we sought to test whether the ERP effects of Plausibility were significant in each tDCS protocol on its own. We used a set of models that were identical to those described above, except that tDCS protocol predictors were removed, as the models were run for each protocol separately.

**Fig. 4:**
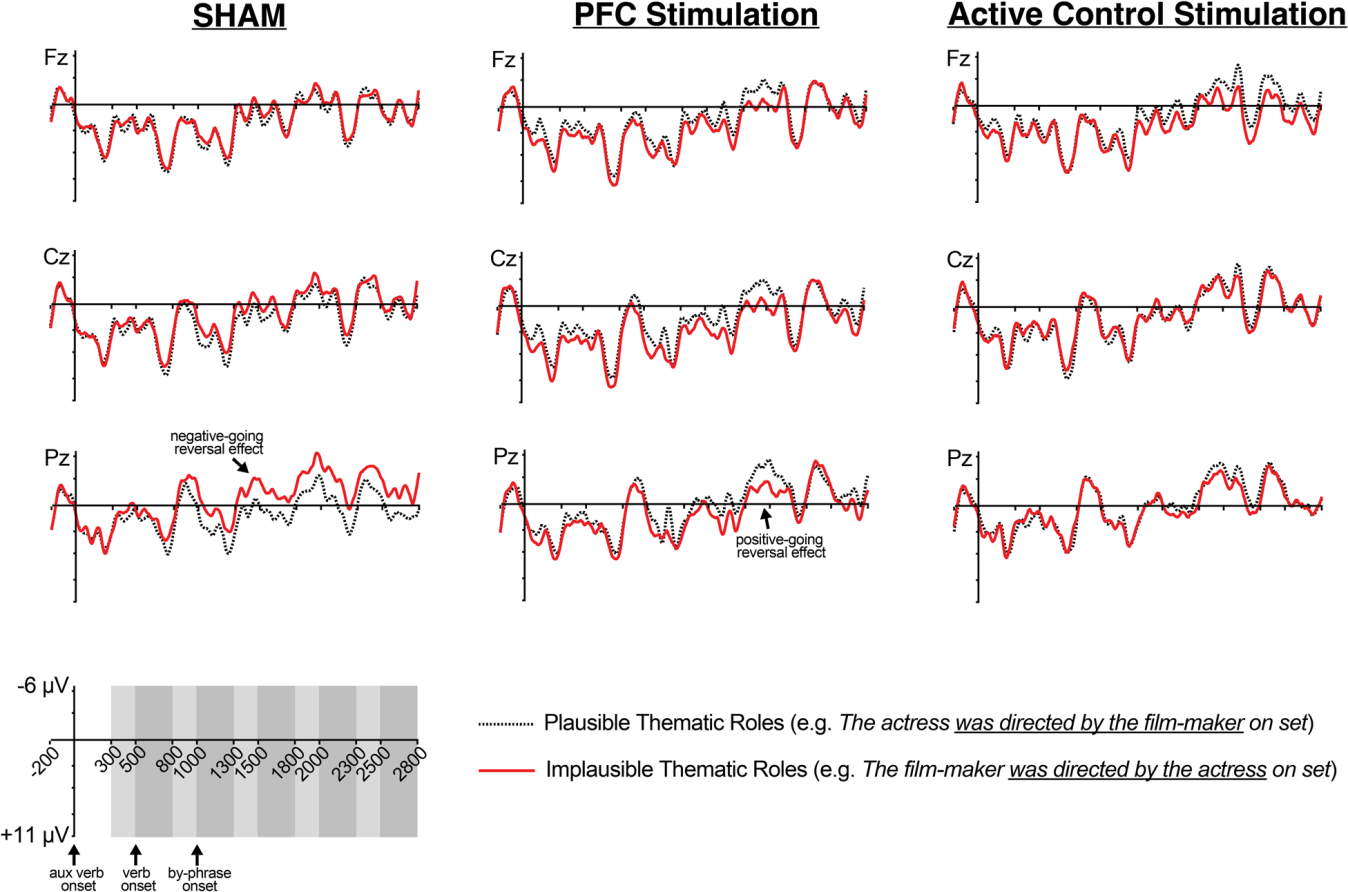
Event-related potentials time-locked to the auxiliary verb and extending through the by-phrase region. Negative is plotted up

**Fig. 5:**
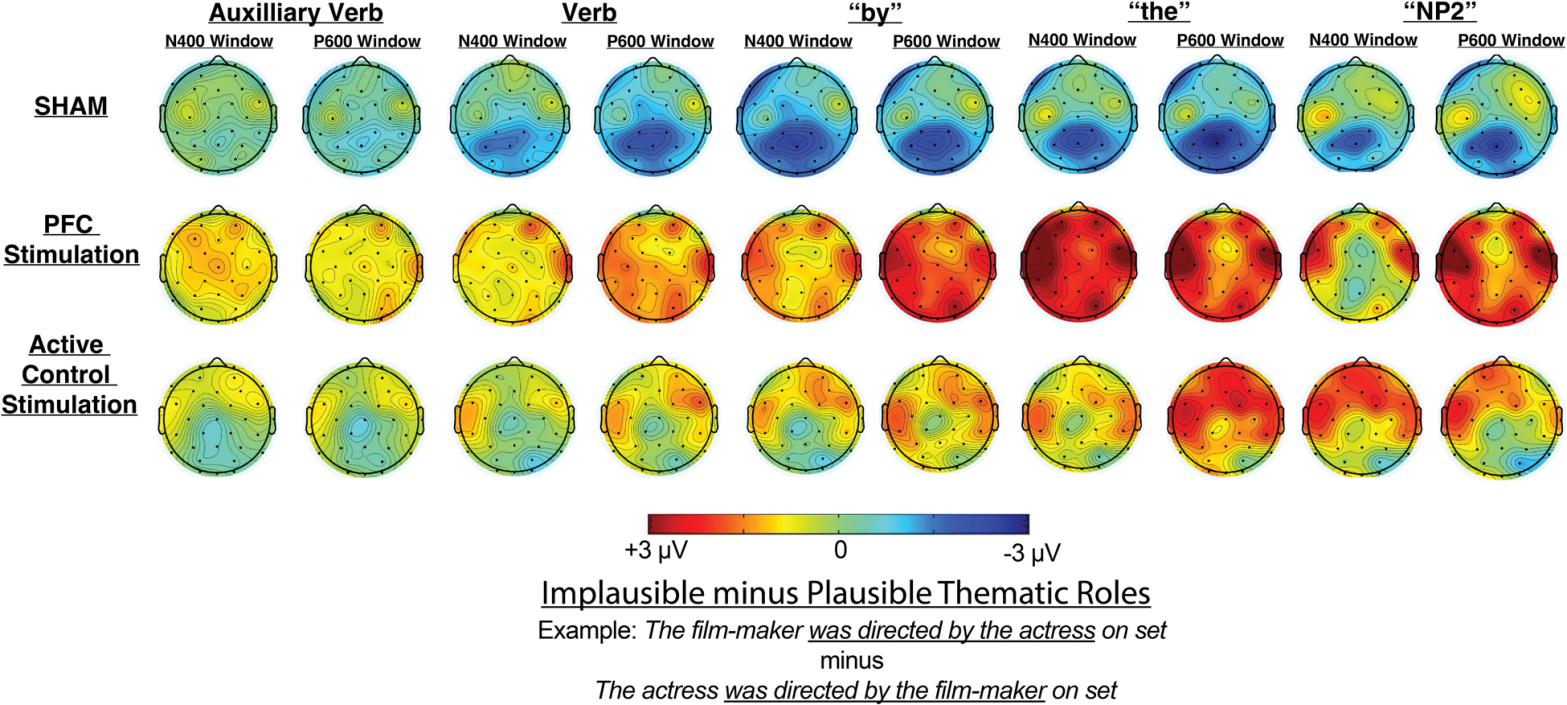
Topographic maps showing the difference in mean amplitude between implausible and plausible conditions for each time window and tDCS protocol tested. The ERPs in the Sham tDCS condition were characterized by negative-going effects with a posterior maximum, while broadly distributed positive-going effects were obtained in the PFC-targeted condition. The Active Control condition also showed positive-going effects, although with a delayed onset, less widely distributed and more limited in duration

As summarized in Table 1, the results showed significant ERP Plausibility effects for each tDCS condition separately (reflected by Plausibility X scalp distribution interactions, meaning that the effects were not equivalent across all electrodes tested). Figures 4 and 5 show ERPs waveforms and topographic distribution maps, respectively, and condition effects following sham stimulation manifested as a lingering negativity that was maximal at posterior electrode sites in response to the implausible thematic roles compared with the plausible condition. In contrast, condition effects following PFC-targeted stimulation were characterized by lingering positive deflections for implausible thematic roles compared with the plausible condition; these effects were fairly widely distributed across the scalp but maximal at left frontal electrode sites. Finally, the active control condition also showed a positive-going effect, although it was smaller, more limited in duration, and less widespread than that observed in the PFC-targeted condition.

## Discussion

The current study used tDCS to test the extent to which an individual’s current PFC state promotes relatively deep processing during language comprehension. Specifically, we examined the ERP response to plausible and implausible thematic role assignment during sentence comprehension, a linguistic manipulation that has served as an important test bed for modern cognitive neuroscience accounts of language processing (Brouwer et al., 2017; Eddine et al., 2024; Kuperberg, 2007; 2020; Li & Futrell, 2024). This is because words, such as “actress” in “the film-maker was directed by the actress,” while slightly implausible or unexpected in context, are also strongly related to the context and revision might render such sentences plausible. A series of influential studies found ERP evidence that such sentences often do trigger revision and updating processes during reading, reflected by P600 effects to this manipulation rather than N400 effects alone (which would be indicative of processing such words as simply semantically unexpected) (Chow et al., 2018; Hoeks et al., 2004; Kim & Osterhout, 2005; Kolk et al., 2003; Kuperberg et al., 2003; Van Herten et al., 2005, 2006). The results of the current study provide a demonstration of variability in how the same readers process this type of sentence, as evidenced by the pattern of ERP results. Specifically, P600 effects of plausibility were found after active tDCS, whereas we observed N400 effects of plausibility after Sham tDCS in the same readers.

This pattern of results suggests that readers tended to treat target words in the implausible condition as simply semantically unexpected after Sham tDCS, consistent with previous work that also found N400 effects in response to this type of linguistic manipulation under conditions that promoted relatively shallow processing (Chow et al., 2018; Kolk et al., 2003; Liao et al., 2022; Van Herten et al., 2006). In contrast, the P600 effects observed in the same readers after PFC tDCS, and to a lesser extent after active control tDCS, suggest that upregulated PFC activity tended to promote revision and updating when readers encountered implausible thematic role assignment, as has also been observed in a number of studies (Chow et al., 2018; Hoeks et al., 2004; Kim & Osterhout, 2005; Kolk et al., 2003; Kuperberg et al., 2003; Van Herten et al., 2005, 2006).

### Implications for psycholinguistic theory

The current set of results have implications for psycholinguistic theory in that they demonstrate first, variability in the language processing approach taken by the same individuals at different times, and second, that this variability was determined by the current state of the PFC. Most theories of language processing do not explicitly account for variability within an individual depending on neurocognitive state and tend to assume “optimal” processing conditions and deep processing as the default. Some theories, such as good-enough parsing accounts, however, have pointed out that this is not always the case; indeed readers do not always “fully” process language and sometimes generate incomplete or incorrect interpretations of the input (Ferreira & Patson, 2007; Ferreira et al., 2002). Other data have also shown that readers/listeners sometimes process language relatively shallowly during periods of inattention or split attention (Boudewyn, 2023; Hubbard & Federmeier, 2021). The current dataset further motivates considering within-individual variability when thinking about language processing, and specifically as related to current neurocognitive state.

### Relation to cognitive control

Our finding that within-participant variation in ERP markers of language processing was determined by stimulation of the PFC, and to a lesser extent, by the neural regions stimulated in the active control condition (which did not fully exclude the PFC; see below for additional discussion) implicates PFC-mediated cognitive control processes in relatively deep linguistic processing. In addition, we found behavioral evidence for increased cognitive control using a well-established nonlinguistic cognitive control task (the DPX) following PFC tDCS compared with either active control or sham tDCS. This set of results builds upon previous recent work that has highlighted the role of cognitive control in language processing generally and specifically in the revision and updating processes thought to be reflected by the P600 and similar late positivities (Brothers et al., 2022; Ness et al., 2023). For example, P600 effects elicited by implausible thematic role assignment have been shown to be correlated with individual differences in performance on cognitive control and working memory tasks (Li et al., 2023; Ye & Zhou, 2008). Furthermore, work on individual differences has found that variation in performance on cognitive control and working memory tasks (which are known to rely heavily on PFC circuits) has been linked to an individual’s tendency to engage in a revision-forward approach, as evidenced by the presence of a P600 rather than an N400 effect in response to implausible thematic roles (Kim et al., 2018; Nakano et al., 2010). The robust late positivities found in the PFC-targeted tDCS condition relative to Sham and Active Control conditions support the idea that PFC-mediated cognitive control processes likely contribute to P600 ERP effects.

However, it is important to note that this does not necessarily imply that the PFC is the direct generator of the P600 itself. While some data implicate PFC regions, such as the left inferior frontal gyrus, as possible generators of the P600, other regions have also been implicated, such as the basal ganglia and fusiform cortex (Brouwer & Hoeks, 2013; Friederici et al., 2003; Frisch et al., 2003; Van de Meerendonk et al., 2011; Wang et al., 2023). Instead, we suggest that PFC-mediated cognitive control promotes deeper processing of incoming words during comprehension by engaging a sequence of processes that ultimately results in robust P600 effects. For example, encountering implausible thematic roles may lead readers to generate multiple competing mental representations based on relatively shallow semantic information (e.g., a sentence in which “breakfast,” “eggs,” and “eat” are involved, and “eggs” are likely to be the object of the verb) and also based on deeper syntactic processing (e.g., despite it being implausible, “for breakfast the eggs would eat” means that the eggs were the agent of the action). The PFC may support both our ability to simultaneously entertain the two interpretations (via working memory mechanisms) and our ability to detect a conflict between the two interpretations (via a cognitive control mechanism) (Botvinick et al., 2004; Carter et al., 1998; Carter & Van Veen, 2007; D’Esposito et al., 2000; Lara & Wallis, 2015, 2015; Veen & Carter, 2006). The detection of conflicting mental representations of the sentence meaning could then trigger a variety of processes in order to resolve the conflict, including reanalysis of the sentence, revision, and updating processes. In other words, the PFC may not be directly involved in producing the P600 waveform itself at the scalp but instead may contribute to the working memory and cognitive control processes that lead to this effect (see Brothers et al., 2022; Wang et al., 2023 for similar accounts).

### Interpretation of active control tDCS results

Finally, while our primary hypotheses concerned the comparison of PFC-targeted tDCS to Sham tDCS, we also included an Active Control condition to be able to evaluate how specific stimulation effects might be to the protocol configuration that targeted the PFC. The results showed that ERP effects in the Active Control condition more closely resembled those found in the PFC-targeted tDCS condition than in the Sham condition in that P600 effects of plausibility were elicited in both active stimulation conditions (although the effects in the PFC condition were significantly earlier, larger, and of longer duration). Interpretation of this finding depends on the extent to which the Active Control served as a “neutral” stimulation montage with respect to the PFC and larger language network. Our modeling of the electric fields produced by each stimulation configuration provides some insight into this question, although it is important to keep in mind that our electric field estimates were produced using a standard brain template (HD-Explore software; soterixmedical.com) and that individual differences in anatomy have a measurable impact on the electric fields produced by tDCS (Laakso et al., 2015, 2016; Mikkonen et al., 2020). Our electric field modeling suggests that the Active Control configuration had the largest influence over centroparietal to occipital regions of cortex and in this respect was distinct from the electric field estimates produced by PFC-targeted stimulation (Fig. 1). However, frontal–temporal and subcortical circuits were likely stimulated by the Active Control tDCS configuration as well (see Supplemental Fig. 1 for additional views of the electric field modeling).

The overlap in the estimated electric fields induced by the Active Control and PFC configurations, as well as the stimulation of language-related frontal–temporal and subcortical regions in the Active Control conditions, may help explain why a similar pattern of results was found in these two conditions. Interestingly, behavioral results from the DPX task (a nonlinguistic cognitive control task) showed an increase in cognitive control performance following PFC stimulation compared with Sham, whereas this benefit was not observed for Active Control stimulation compared to Sham. This further supports the interpretation that PFC tDCS specifically stimulated cognitive control circuits, and that the similarities between PFC tDCS and Active Control tDCS on the language task may best be attributed to stimulation of the language network in the Active Control condition.

## Conclusions

The results of this study have implications for psycholinguistic theory, particularly about the extent to which the prefrontal cortex (PFC) contributes to revision and updating processes during language comprehension. We obtained a qualitatively different pattern of ERP results when the same readers encountered implausible compared to plausible thematic roles after PFC-targeted stimulation, and to a lesser extent, after Active Control stimulation, compared with sham neurostimulation. Implausible thematic roles appeared to be processed as semantic anomalies following sham stimulation, whereas readers appeared to be more likely to detect linguistic conflict and engage in revision and updating processes when in a state of heightened PFC engagement. These results provide a novel demonstration for variability *within* an individual in how language is processed depending on current neurocognitive state and provide support for the role of PFC circuits in revision and updating processes during language comprehension.

## Data Availability

The data reported here is available through the Open Science Framework: https://osf.io/mbwhz/.

## Code availability

Key scripts used in analyzing the data reported here are available through the Open Science Framework: https://osf.io/mbwhz/.

## Declarations

### Open practices statement

The data reported here is available through the Open Science Framework: https://osf.io/mbwhz/. This experiment was not pre-registered.
